# Inclusive instructional communication in higher education: A comparative policy analysis of South Africa and Malawi

**DOI:** 10.4102/sajcd.v73i1.1185

**Published:** 2026-07-21

**Authors:** Ben de Souza

**Affiliations:** 1 Department of Secondary and Post-School Education, Faculty of Education, Rhodes University, Makhanda, South Africa

**Keywords:** comparative policy analysis, inclusive communication, communication disorders, higher education, South Africa, Malawi

## Abstract

**Background:**

Inclusive instructional communication is necessary for equitable participation of students with communication disorders in higher education. However, limited evidence exists on how policy and legal frameworks in Southern Africa address communicative access, multilingual teaching and support for students with communication disorders.

**Objectives:**

This study examined how selected frameworks in South Africa and Malawi represent inclusive instructional communication for students with communication disorders.

**Method:**

A comparative policy analysis was conducted using qualitative content analysis in an integrated critical realist framework. Ten documents were analysed, focusing on inclusivity, instructional communication, multilingualism, disability support and digital accessibility.

**Results:**

The analysed frameworks express commitments to equity, inclusivity and participation in higher education. South Africa gives attention to multilingualism and language access, while Malawi addresses broader inclusivity. However, the analysis identified limited guidance on instructional communication practices for students with communication disorders. The frameworks did not clearly specify lecturer training in inclusive communication, multimodal teaching approaches, sign language integration, augmentative and alternative communication support or accessible digital learning practices.

**Conclusion:**

The absence of explicit policy provisions for educator preparation, accessible communication practices and communication-support technologies may limit practical guidance on epistemological inclusion of students with communication disorders.

**Contribution:**

This study demonstrates how frameworks in South Africa and Malawi insufficiently address inclusive instructional communication in higher education, arguing for policies that include communication-focused lecturer development, multilingual and multimodal teaching practices, accessible digital learning environments and student support.

## Introduction

Instructional communication is a critical determinant of effective teaching and learning in higher education because it shapes how knowledge is transmitted, interpreted and experienced by students (Morreale, 2015). In communication research, instructional communication refers to the systematic study of how messages, relational dynamics and communicative strategies influence learning processes and educational outcomes (Goodboy, 2018; Sellnow et al., 2015). Conley and Ah Yun (2017) summarised that:

[*I*]nstructional communication has since been generally defined as the area of research that investigates the communicative dynamics of teaching and learning relative to the exchange of meanings between, and among teachers and students, situated in any context or setting, about any subject matter, of any field. (p. 452)

In this sense, the relevance of instructional communication is evident in contexts characterised by communicative diversity, where students differ in language proficiency, cultural background and communicative abilities (Powell & Powell, 2016). In such contexts, equitable participation in learning depends not only on physical access to institutions but also on communicative access to teaching, interaction and assessment processes. In Australian context, Cronin et al. (2020) observed that for students with communication disorders, communicative access is directly linked to academic participation and learning outcomes. Globally, examples have been given from research that students who are deaf or hard of hearing may experience difficulties with speech perception in lecture environments, classroom discussion participation, auditory processing during rapid instruction and access to spoken information in multilingual settings (Bell et al., 2016; Percival et al., 2025). Students with speech, language and cognitive-communication disorders may also encounter barriers in oral presentations, interactive classroom engagement, comprehension of academic discourse and assessment communication (Kormos & Smith, 2023). As Roberts (2026) found in the United Kingdom (UK) higher education context, these barriers are intensified in higher education environments where communication practices are heavily dependent on verbal instruction, lecture-based teaching and text-dense learning materials. He concluded that these ‘are not only technical barriers but institutionalised denials of epistemic credibility and participatory belonging’ (Roberts, 2026, p. 18). Research in audiology and communication disorders has further shown that inaccessible instructional environments may contribute to social isolation, reduced classroom participation and poorer academic adjustment among students with communication disorders (Bell et al., 2016; Bell & Swart, 2018; Percival et al., 2025).

In Southern Africa, higher education systems operate in historically stratified societies shaped by colonial legacies, linguistic hierarchies and socio-economic inequalities. Countries such as South Africa and Malawi have adopted policy and legal frameworks intended to promote equity, inclusion and redress in higher education. However, these frameworks have mainly focused on structural dimensions of access, including enrolment expansion, financial support and physical accessibility (De Souza & Kathumba, 2026; Mabokela & Mlambo, 2017). Comparatively limited attention has been given to the communicative processes through which teaching and learning occur. This limitation has implications for students with communication disorders and related disabilities who may require captioning, sign language interpretation, augmentative and alternative communication (AAC), accessible digital platforms, multimodal teaching practices and adapted interactional approaches to participate effectively in higher education.

Available evidence from South Africa indicates that students with hearing impairments continue to experience barriers to participation and support in higher education despite inclusive policy commitments (Bell et al., 2016). Research has also highlighted communication barriers linked to inadequate South African Sign Language proficiency among educators and limited communication support practices in educational settings (Ndwandwe & Joseph, 2025). Although comparable higher education research from Malawi remains limited, studies on multilingualism and educational participation suggest that students may experience reduced comprehension and participation when instructional communication practices are not adapted to linguistic diversity (Chimpololo, 2024). Furthermore, multilingualism is a defining feature of both South African and Malawian classrooms, where many students learn or process information through second or additional languages (Chimpololo, 2024; Heugh, 2013). In such settings, communication barriers extend beyond disability and include linguistic and cultural factors that influence comprehension, participation and interaction. Students with communication disorders may therefore experience layered barriers associated with hearing, speech, language, auditory processing, literacy or multimodal communication access. Without intentional instructional communication strategies, these barriers may contribute to reduced classroom participation, academic exclusion and unequal learning opportunities. Therefore, there is a need to examine how policy environments facilitate or constrain communicative access. This is important in higher education where communicative competence is key to learning, participation and assessment.

### Aim and objectives

This study aimed to examine how inclusive instructional communication is conceptualised in policy and legal frameworks in South Africa and Malawi. The specific objectives were to:

- Analyse national policy documents to identify how instructional communication and inclusion were represented
- Examine the extent to which policies addressed the communicative needs of students with communication disorders, including auditory, linguistic, multimodal and digital communication barriers
- Compare similarities and differences between South African and Malawian policy frameworks concerning inclusive instructional communication.

## Literature review

### Instructional communication and inclusion in higher education

Instructional communication has long been recognised as central to teaching and learning in higher education, particularly in relation to how meaning is constructed, negotiated and understood in classroom environments (Morreale, 2015). Communication in instructional settings is not simply the transmission of information but a relational and representational process through which learning is mediated (Goodboy, 2018). Research in this field has examined constructs such as clarity, immediacy, credibility and feedback, demonstrating their influence on student motivation, participation and academic outcomes (Houser, 2019). However, much of this research has been conducted in North American and European higher education contexts that often assume relatively homogeneous student populations and stable communicative norms (Sellnow et al., 2015). As a result, instructional communication research has paid limited attention to communicative accessibility for students with disabilities, multilingual students and students who rely on alternative communication modalities.

Communication disorders research, on the other hand, has demonstrated that students with hearing, speech, language and cognitive-communication impairments may experience barriers related to listening effort, speech perception, note-taking, verbal participation and processing of spoken academic discourse (Kormos & Smith, 2023). Research from South Africa has shown that students who are deaf or hard of hearing often experience communication barriers linked to inadequate support services, limited disclosure practices and inaccessible instructional environments (Bell et al., 2016). Similarly, Percival et al. (2025) found that communicative, instructional and environmental factors significantly influence the adjustment of deaf and hard-of-hearing students in higher education settings. Their findings highlighted the importance of accessible communication practices, institutional support and lecturer awareness in promoting equitable participation.

Inclusive education discourse advocates the removal of barriers to learning and participation through adaptation of educational systems and practices (Liasidou, 2012). In this regard, Maguvhe and Masuku (2023) argued that ‘[a]s Africa is confronted with unique inclusive education challenges, concerns and issues, this needs an African epistemology intervention since African epistemology precisely deals with African issues’ (p. 2). In higher education, inclusion has largely been framed through commitments to access, equity and student support. However, the communicative dimensions of inclusion, including how teaching is delivered, how interaction occurs and how students access classroom discourse, have received comparatively less attention in policy and research. This study brings instructional communication and communication disorders discourse into dialogue, highlighting the need to conceptualise communication as a central mechanism of inclusion. Inclusive instructional communication involves the use of communication practices that facilitate equitable participation in teaching and learning (Ledbetter, 2023). These practices include multimodal teaching approaches, accessible digital communication, visual learning supports, sign language interpretation, captioning, AAC integration, adapted feedback processes and linguistically responsive teaching strategies. For students with communication disorders, such practices are fundamental to participation and learning.

### Linguistic diversity, disability and communication access

In (South) African higher education contexts, instructional communication is closely linked to linguistic diversity and historical power relations (Steyn & Reygan, 2017). Multilingualism is a defining feature of Southern African classrooms, yet colonial languages (including English) continue to dominate as the principal language of teaching and learning (Kondowe et al., 2024). This dominance reflects historical and colonial language hierarchies that continue to shape access to knowledge and academic participation (Heugh, 2015). Leki (2007) observed that students who learn or process information through a second or additional language may experience difficulties related to comprehension, classroom participation and academic discourse. For students with communication disorders, these challenges may be compounded by auditory processing demands, speech perception difficulties, reduced access to spoken information and communication fatigue (Kormos & Smith, 2023). Percival et al. (2025) exemplified that deaf and hard-of-hearing students, for example, may struggle to follow spoken lectures delivered rapidly in English, especially in contexts where captioning, note-taking support or sign language interpretation are unavailable.

Research in communication disorders, deaf education and neurodiversity has demonstrated that communication barriers in educational settings are linked to reduced participation, limited interaction and poorer educational experiences (Ndwandwe & Joseph, 2025; Shoko, 2024a, 2024b). These studies have also shown that inadequate lecturer proficiency in sign language, limited communication support and inaccessible classroom communication practices can restrict meaningful educational participation for deaf learners. Despite these realities, higher education policy and research in many African contexts continue to address disability inclusion and multilingualism as separate issues. This separation limits the development of integrated approaches to communicative access. Policies may promote multilingualism without addressing disability-related communication access, or support disability inclusion without specifying how instructional communication practices should be adapted in multilingual environments. Consequently, communication remains insufficiently recognised as a structural component of educational inclusion.

### Integrated theoretical framework for the study

This study was informed by critical realism and drew conceptually on instructional communication, inclusive education and decolonial perspectives. The framework was used to guide both the coding process and interpretation of findings. Bhaskar’s critical realism argues that reality exists independently of human perception, but our knowledge about it is always socially produced and fallible. He maintains that deeper underlying structures and mechanisms shape observable events, and that critical inquiry should expose these structures in order to support human emancipation and social transformation. As Bhaskar (2016) explained, ‘the world contains not only specific causal laws but causality as such’ (p. 3). Bhaskar (2008, 2016) distinguished between three domains of reality: the empirical (what is observed), the actual (events that occur) and the real (underlying structures and mechanisms that shape observable events). In this study, the empirical domain referred to explicit statements contained in policy documents. The actual domain concerned how policies may shape communicative practices in higher education settings. The real domain referred to underlying policy assumptions, institutional structures and power relations that influence communicative inclusion or exclusion.

Critical realism guided the coding process by focusing analysis on three interconnected areas: (1) explicit references to inclusion, communication and disability; (2) policy mechanisms intended to support implementation, such as lecturer development, multilingual strategies and digital accessibility; and (3) policy omissions or silences related to communicative access for students with communication disorders. Critical realism also guided interpretation by enabling analysis beyond policy rhetoric to examine whether policies identified practical mechanisms capable of supporting inclusive instructional communication.

Instructional communication theory provided the analytical basis for examining how teaching and learning are mediated through communication practices (Goodboy, 2018; Sellnow et al., 2015). In this study, instructional communication included language of instruction, multimodal teaching approaches, classroom interaction, feedback processes and digital communication practices. The theory was particularly relevant for examining whether policies addressed the communicative conditions necessary for equitable participation by students with communication disorders.

Inclusive education perspectives contributed to focus on removal of barriers to participation and access for students with disabilities (Liasidou, 2012). In this study, communicative barriers included auditory barriers, linguistic barriers, inaccessible digital communication, limited sign language provision and exclusionary interactional practices. Disability was therefore understood not only as an individual characteristic but also as a product of inaccessible educational environments and communication systems (Zhou, 2023).

Decolonial theory challenges the dominance of colonial knowledge systems and seeks to centre indigenous, local and historically marginalised ways of knowing in education, research and institutional practice. In higher education, it calls for curricula, teaching and knowledge production that reflect African realities, languages and epistemologies. Ndlovu-Gatsheni (2018) argued that decolonisation is about achieving ‘epistemic freedom’ through the dismantling of colonial patterns of knowledge and power. Decolonial perspectives informed the analysis by highlighting how dominant educational systems prefer particular languages, communication practices and forms of knowledge (Heleta, 2016). This perspective was relevant for examining how higher education policies addressed multilingualism, language access and communicative participation in historically unequal educational contexts.

Bringing these philosophical and theoretical perspectives together, the study conceptualised inclusive instructional communication as a policy and pedagogical process through which diverse communicative needs are accommodated in teaching and learning environments. This includes attention to multilingual communication, multimodal teaching, accessible digital learning, sign language support, AAC inclusion and communication-responsive lecturer practices.

## Research methods and design

### Study design

This study adopted a qualitative comparative policy analysis design. Bingham et al. (2019) explained that ‘[q]ualitative comparative analysis (QCA) is a case-oriented research method designed to identify causal relationships between variables and a particular outcome’ (p. 1). They concluded that:

[*P*]olicy analysts could employ QCA to examine educational policy to compare policy programs or determine causal combinations that impact policy implementation or outcomes. The nature of QCA generates deterministic results which align with the nature of policy analysis. (Bingham et al., 2019, p. 12)

As such, the design was appropriate because the study examined how inclusive instructional communication is represented across policy texts in South Africa and Malawi. A qualitative approach allowed close analysis of meanings, assumptions, omissions and implementation provisions in policy documents, while the comparative dimension enabled the identification of similarities and differences.

As indicated in the last section, the study was philosophically and metatheoretically informed by critical realism (Bhaskar, 2008, 2016). Bhaskar (2016) explained that critical realism enables the method of ‘immanent critique’ (p. 1). He explained further that:

[*I*]mmanent critique is an essential part of the method of critical realist philosophy. It specifies that criticism of an idea or a system should be internal, that is, involve presuppositions and origins of CR 3 something intrinsic to what (or the person who) is being criticised. It typically identifies a theory/practice inconsistency, showing that the position being disputed involves a claim or analysis that would undermine the point, values or substance of the position; so that it undermines or ‘deconstructs’ itself. A moment’s reflection shows indeed that this is the only way an argument can ever ultimately be won. Merely to assert what one believes will get nowhere unless it impinges in some way on what one’s opponent believes (Bhaskar, 2016, pp. 2–3).

In immanent critique methodological terms, critical realism guided the analysis at three levels. Firstly, the study examined the *empirical* level by identifying explicit policy statements on inclusivity, language, disability, teaching and learning, digital access and communication. Secondly, it examined the *actual* level by considering the implementation provisions through which policies proposed to support inclusive practice. Thirdly, it examined the *real* level by interpreting policy silences and structural mechanisms that may enable or constrain inclusive instructional communication.

### Setting

The study was situated in the higher education policy environments of South Africa and Malawi. These countries were selected because they share a Southern African regional context and multilingual educational histories, while differing in higher education system size, policy development and institutional capacity. South Africa has a larger and more differentiated post-school education system comprising traditional universities, universities of technology, and technical and vocational education and training colleges (Cloete, 2014). Malawi has a smaller higher education system that is shaped by expansion, quality assurance, national development priorities and resource constraints (Kadzamira, 2020). Both contexts are relevant to this study because they have policy commitments to equity, inclusivity, disability support, language access and digital participation. These policy areas are directly connected to inclusive instructional communication, especially for students with hearing, speech, language and cognitive-communication disabilities.

### Study documents and sampling strategy

The study documents comprised national policy and legal documents relevant to higher education, teaching and learning, language, disability inclusion and digital access in South Africa and Malawi. A purposive sampling strategy was used to select policy documents that were directly aligned with the study aim and objectives. Purposive sampling in document or policy analysis research is a non-probability sampling technique in which researchers intentionally select documents, policies or cases that are most relevant to the research question or goal and likely to provide rich and meaningful data. As Suri (2011) explained, the rationale of purposive sampling is found in the ability to surface relevant information in much detail. The inclusion criteria were as follows: (1) the document had to be an official national policy, Act, strategy or framework; (2) it had to address higher education directly or include provisions relevant to teaching, learning, disability, language, communication or digital access; (3) it had to be publicly accessible; and (4) it had to have relevance to inclusive instructional communication. Documents were excluded if they were institutional policies from a single university, draft documents without clear official status, documents focused only on basic education with no relevance to higher education, or documents that had no substantive relevance to communication, disability, language or teaching and learning.

Recognising that inclusive instructional communication spans several policy areas, the dataset included five documents per country. For South Africa, the analysis included policies on higher education transformation, language, disability and digital access: (1) *White Paper for Post-School Education and Training*, (2) *Language Policy for Higher Education*, (3) *Language Policy Framework for Public Higher Education Institutions*, (4) *Strategic Policy Framework on Disability for the Post-School Education and Training System* and (5) *National Integrated Information and Communication Technology (ICT) Policy White Paper*. For Malawi, the analysis included national education, higher education, inclusive education, disability and ICT policy documents: (1) *National Education Policy*, (2) *National Education Sector Investment Plan 2020–2030*, (3) *National ICT Policy*, (4) *Persons with Disabilities Act, 2024* and (5) higher education standards issued through the National Council for Higher Education (NCHE). Malawi does not have a dedicated higher education language policy comparable to South Africa’s higher education language policy framework.

As Suri (2011) emphasised, this sample was selected for information richness. Information richness was understood as the extent to which each document contributed relevant content on one or more dimensions of inclusive instructional communication, namely language access, disability inclusion, teaching and learning, digital access, lecturer support, assistive technologies or implementation mechanisms. The sample was not intended to represent all higher education policies in both countries. Its purpose was to provide a focused and policy-relevant basis for examining how inclusive instructional communication is addressed across intersecting national frameworks. Documents were identified through official government websites, policy repositories and recognised public policy databases. South African documents were sourced from the Department of Higher Education and Training (DHET) and the South African Government portal. Malawian documents were sourced from the Government of Malawi, the Ministry of Education (MoE), the NCHE, the Malawi Communications Regulatory Authority, United Nations Children’s Fund Malawi and recognised policy repositories hosting official education and disability documents.

### Data generation

Data were generated through qualitative document analysis. According to Karppinen and Moe (2019), qualitative document analysis is a research method that systematically examines and interprets written, visual or digital documents to identify patterns, meanings, themes and social or policy contexts relevant to a research question or aim. The data sources were official policy documents written in English. Document analysis was appropriate because the study examined how inclusive instructional communication is formally represented in policy texts. A structured document review protocol was developed to guide extraction of relevant content. The protocol included the following categories: (1) inclusivity and equity; (2) disability and communication disorders; (3) language and multilingualism; (4) teaching, learning and assessment; (5) digital access and assistive technologies; (6) lecturer training and professional development; (7) implementation responsibilities; and (8) monitoring and accountability. Each document was read in full before coding. The coding done here was not a quantitative one. This is based on what Rapley and Rees (2018) explained that:

[*R*]esearchers gather specific documents, then establish a coding frame and apply that coding frame to the documents to count the number of times particular words, phrases or themes are used. Clearly, this enables descriptive and statistical findings to be established. However, this is often seen by some qualitative researchers as a relatively limited, albeit systematic approach, in that it predefines the key aspects of analysis alongside removing words and phrases from their immediate and broader context. (p. 379).

Therefore, this was a qualitative coding, whereby relevant excerpts were extracted into a document matrix organised by country, document title, policy focus, relevant provisions, communication-related content and implementation mechanisms. This enabled comparison within and across countries.

### Data analysis

Data analysis was conducted using qualitative content analysis, combining deductive and inductive coding. Qualitative content analysis is a systematic research method used to interpret and categorise textual, visual or audio data by identifying themes, patterns and meanings within the content (Selvi, 2019). In policy research, deductive coding involves analysing data using pre-established themes or theoretical categories, whereas inductive coding involves generating themes and categories directly from the data as patterns and meanings emerge (Calvo & De la Cova, 2023). Rapley and Rees (2018) observed that:

[*A*]nalytic work on and with documents can be loosely divided into two areas: work that focuses on the actual textual and extra-textual content of documents; and work that focuses on some aspect of the use, role and function of documents in everyday and organisational settings. (p. 378)

This study moved between the two areas through the deductive and inductive coding. Deductive codes were developed from the study objectives and theoretical framework. These included instructional communication, disability inclusion, auditory access, linguistic access, multimodal communication, digital accessibility, assistive technology, lecturer development, implementation mechanisms and policy accountability. Inductive coding was then used to identify themes that emerged from the policy texts. Examples included broad inclusion without communication-specific guidance, language access framed mainly through multilingualism, disability addressed through support services, and digital access addressed as infrastructure with limited attention to communication accessibility.

Coding was conducted manually. A coding table was created in which all extracted policy excerpts were linked to document title, country, code, theme and interpretive note. Codes were refined through repeated reading of the documents. Similar codes were grouped into broader themes, and themes were compared across the South African and Malawian documents. Critical realism, using the immanent critique method (Bhaskar, 2016), informed the interpretation in a concrete way. Explicit policy provisions were taken as empirical data. Implementation arrangements, such as lecturer development, funding, monitoring, accessibility standards and institutional responsibilities, were taken as possible mechanisms for change. Policy silences, such as lack of reference to captioning, sign language, AAC, accessible digital teaching or auditory access, were interpreted as possible gaps in the mechanisms needed to support students with communication disorders.

To strengthen rigour, an audit trail was maintained. This included the document list, inclusion and exclusion decisions, coding categories, extracted excerpts, coding notes and theme development records. Reflexive notes were also kept to monitor possible interpretation bias, especially where policy documents used broad terms such as inclusivity, access or participation without specifying communication-related support. Self-reflexivity was used during theme refinement, with attention to whether interpretations remained grounded in the policy texts. The analysis avoided making claims beyond the scope of the documents and took findings as policy-based indicative insights. This was also in tandem with principles of critical realism because:

[*I*]t is important to note that the method of immanent critique prohibits any simple-minded or unmediated transfer of results from one context to another. There must always be an independent analysis of the new domain before the possibility of any transapplication can be considered. (Bhaskar, 2016, p. 8)

### Ethical considerations

Whether or not research involves human participants, ethical considerations remain part of the process. Accordingly, ethical principles were carefully observed in this policy analysis study. Formal ethical approval was not required because the research relied exclusively on publicly available policy documents and did not involve human participants, confidential information or personal data. Consistent with guidance by Bowen (2009), who noted that document analysis requires attention to authenticity, credibility, representativeness and meaning, ethical integrity was maintained through the use of official and publicly accessible documents, accurate recording of policy details, faithful representation of policy provisions, and avoidance of selective reporting. To minimise interpretation bias, the analysis systematically compared policy intentions, implementation provisions and policy silences across all included documents.

## Results

The findings are presented in relation to the study objectives, with specific attention to how inclusive instructional communication for students with communication disorders is represented in South African and Malawian policy and legal frameworks. The analysis generated five themes: (1) policy commitments to access and inclusivity; (2) disability, communication disorders and communicative participation; (3) multilingualism and language access; (4) digital technologies and multimodal communication; and (5) staff development and implementation mechanisms.

### Policy commitments to access and inclusivity

Across both countries, policy documents express strong commitments to equity, access and inclusivity. In South Africa, the *White Paper for Post-School Education and Training* frames the post-school system as a means of building ‘a fair, equitable, non-racial, non-sexist and democratic South Africa’ (South Africa. DHET, 2013, p. xi). It further states that the right to access an educational institution ‘is not enough’ and that institutions must provide education of ‘high quality’ (South Africa. DHET, 2013, p. viii). The South African *Strategic Policy Framework on Disability for the Post-School Education and Training System* provides a stronger disability-specific basis for inclusivity. It states that:

[*T*]he needs of people with disabilities are not fully addressed in the context of post-school education and training [*and that these needs should be approached from a*] human rights and social developmental perspective. (South Africa. DHET, 2018, p. 12)

The same framework aims to create:

[*A*]n enabling and empowering environment across the PSET system [*and to set*] norms and standards for the inclusion of students and staff with disabilities in all aspects of university, college and skills development life. (South Africa. DHET, 2018, p. 14)

In Malawi, the *National Education Policy* similarly positions education as central to national development and states that it seeks to provide quality education through ‘expanded access and equity, improved quality and relevance, and improved governance and management’ (Malawi. Ministry of Education, Science and Technology [MoEST], 2016, p. iii ‘Foreword’). In higher education, the policy recognises that ‘those who are physically challenged are very few in the system’ and identifies ‘insufficient qualified staff’, ‘inadequate teaching and learning resources’ and ‘inadequate investment in infrastructure and rehabilitation’ as challenges (Malawi. MoEST, 2016, p. 25). Malawi’s *National Education Sector Investment Plan 2020–2030* also identifies higher education access as a priority and calls for expansion of ‘learning space in Higher Education, including virtual learning space [with attention to] disadvantaged students and females’ (Malawi. MoE, 2020, p. 46). It also calls for ‘inclusive education facilities’ and lecturer capacity-building in ‘pedagogical skills’ (Malawi. MoE, 2020, pp. 46–47). These commitments show that both countries recognise access, equity and disability inclusivity. The key gap is that inclusivity is generally framed at the level of institutional access, infrastructure, governance and student support, with less specification of how teaching, learning and assessment communication should be made accessible to students with hearing, speech, language or cognitive-communication disabilities.

### Disability, communication disorders and communicative participation

The South African disability framework provides the clearest policy basis for connecting disability inclusion to teaching and learning. The *White Paper for Post-School Education and Training* states that a strategic disability framework should take into account ‘the multiple types of disabilities that exist [and] the differentiated response required by the post-school system’ (South Africa. DHET, 2013, p. 46). It further states that reasonable accommodation should include:

[*T*]he built environment as well as the use of specialised technology and assistive devices [*and that*] a more integrated approach to adapting teaching and learning methodologies and approaches is necessary. (South Africa. DHET, 2013, p. 47)

This is directly relevant to students with communication disorders because teaching and learning methodologies include the communication conditions through which lectures, discussions, assessments, tutorials and feedback are accessed. For deaf and hard-of-hearing students, this may include captioning, sign language interpreting, assistive listening systems, lecture recordings and visual support. For students who use AAC, it may include communication-compatible platforms, flexible participation formats and assessment accommodations. The South African *Strategic Policy Framework on Disability* also defines reasonable accommodation as including ‘assistive devices and technology’ and states that people with disabilities should not be excluded from participation where equal enjoyment is available (South Africa. DHET, 2018, p. x). It also recognises ‘note-takers [and] interpreters for deaf-blind persons’ as forms of personal assistance (South Africa. DHET, 2018, p. x). These provisions offer an important foundation for communicative access, but the policy does not develop a detailed higher education communication-access standard for students with communication disorders.

In Malawi, the *Persons with Disabilities Act, 2024*, provides the strongest communication-specific legal language. It defines ‘communication’ as disseminating information in a format that is ‘accessible and comprehensible’ and includes ‘display of text, braille, tactile communication, sign language, signs, large print, accessible multimedia … [*and*] augmentative or alternative modes’ (Malawi. Parliament of Malawi [PoM], 2024, p. 3). It also defines ‘assistive products’ as aids, appliances and technologies that support functioning and defines disability as impairment interacting with barriers that may hinder ‘full and effective participation in society’ (Malawi. PoM, 2024, p. 4). This definition is highly relevant to inclusive instructional communication because it explicitly includes AAC, sign language, accessible multimedia and plain language. It provides a strong legal basis for higher education institutions to make classroom communication, digital learning platforms, learning materials and assessments accessible. Nevertheless, the Act does not specify how these communication rights should be translated into (higher education) teaching and learning practices. The *Minimum Standards for Higher Education Institutions in Malawi* address special needs access mainly through physical facilities and learning resources. The standards require that ‘all buildings shall provide for special needs access’ and that higher education institutions should ensure that building design accommodates ‘universal access’ (Malawi. NCHE, 2015, p. 23). The same document also states that library and learning resource centres should provide resources ‘suitable to people with special needs’ (Malawi. NCHE, 2015, p. 24). These standards are useful, but they remain more focused on physical and resource access. They do not specify captioning, interpreter services, AAC access, classroom acoustics, speech-to-text support, accessible online assessment or lecturer training in communication-accessible teaching.

### Multilingualism and language access

Language policy was more developed in South Africa than in Malawi. The South African *Language Policy for Higher Education* recognises that ‘[*e*]veryone has the right to receive education in the official language or languages of their choice in public educational institutions where that education is reasonably practicable’ (South Africa. MoE, 2002, p. 3). The 2020 *Language Policy Framework for Public Higher Education Institutions* builds on this by stating that it aims to strengthen indigenous languages as languages of ‘scholarship, teaching and learning and communication’ (South Africa. DHET, 2020, p. 10). The 2020 framework also states that multilingualism should ‘facilitate meaningful access and participation by university communities (students and staff)’ and support ‘cognitive and intellectual development’ (South Africa. DHET, 2020, p. 5). It therefore provides a strong policy basis for language access in higher education. Meanwhile, the framework acknowledges earlier implementation weaknesses, including ‘lack of enforceable mechanisms’, ‘lack of funding or incentives’ and ‘lack of clear directives … on how multilingualism is to be realised within higher education institutions’ (South Africa. DHET, 2020, p. 10). This acknowledgement is important because it shows that policy recognition of multilingualism has not automatically produced classroom-level language access. For students with communication disorders, this matters because multilingual classrooms can intensify listening, speech perception, language processing and comprehension demands. A deaf or hard-of-hearing student, a student with a language disorder or a student using AAC may face added barriers when academic teaching relies on rapid spoken English without visual reinforcement, captioning, sign language access or multilingual scaffolding.

In Malawi, there is no equivalent higher education language policy. The *National Education Policy* addresses higher education access and quality but does not provide a higher education language framework. Its discussion of higher education identifies access, quality, relevance and capacity development as priorities, including ‘equitable access to quality and relevant special and inclusive higher education’ (Malawi. MoEST, 2016, p. 25). However, it does not specify how language of teaching and learning in higher education should support students with communication disorders or multilingual learners. This indicates that South Africa has more developed language policy architecture, while Malawi has broader education policy commitments without detailed higher education language provisions. In both cases, language access is not fully integrated with communication disability access.

### Digital technologies and multimodal communication

Both countries recognise ICT as central to education, access and participation. South Africa’s *National Integrated ICT Policy White Paper* states that universal access and service aim to ensure that all South Africans can access basic communication services and that, ‘in the digital era, access to the Internet and the services and content carried on this platform becomes necessary and is therefore considered “basic”’ (South Africa. Department of Telecommunications and Postal Services [DTPS], 2016, p. 26). It also frames digital access as part of participation and emphasises that:

[*D*]igital and mobile infrastructure and technologies are tools to enable all citizens to engage with, create and access information and services from a range of sources anywhere and at any time. This assists in addressing unequal access to public and other services (such as education and healthcare) and boosts opportunities for employment and economic growth. Digital transformation is by its very nature disruptive and has and will continue to change the way everyone communicates, interacts and transacts (South Africa. DTPS, 2016, p. 109).

Malawi’s *National ICT Policy* defines ICT broadly as technologies including ‘computers, telecommunication, audio-visual systems, and postal systems’ that enable information and communication services (Malawi. Government of Malawi [GoM], 2013, p. v). It also states that government will integrate ICTs in education ‘at all levels in order to improve access to and quality of education’ (Malawi. GoM, 2013, p. 8). Malawi’s *National Education Policy* identifies open and distance learning challenges, including ‘poor and inadequate information and communication technology (ICT) infrastructure’, ‘limited, unreliable and expensive internet connectivity or facilities’ and ‘inability to provide inclusive education’ (Malawi. MoEST, 2016, p. 26). The *National Education Sector Investment Plan* also calls for ‘virtual learning space’ in higher education (Malawi. MoE, 2020, p. 46). These policy provisions show that ICT is seen as a mechanism for widening access. The limitation is that ICT access is rarely connected to communication accessibility for students with communication disorders. Policies do not provide detailed guidance on captioned video lectures, speech-to-text tools, accessible multimedia, AAC-compatible learning management systems, sign language in online learning, hearing-assistive technologies or accessible virtual assessment. This is an important gap because students with communication disorders may require digital environments that support visual, auditory, tactile, linguistic and alternative communication modes.

### Staff development and implementation mechanisms

Both countries recognise staff development and implementation capacity, but neither provides sufficient detail on lecturer competence in inclusive instructional communication. In South Africa, the *White Paper for Post-School Education and Training* states that awareness of the needs of students and staff with disabilities should be built ‘alongside the capacity to address disability at all levels of post-school institutions, including lecturers, support staff and management’ (South Africa. DHET, 2013, p. 47). This is relevant because it connects disability inclusion to lecturer and staff capacity. The statement supports the need for lecturer training in communication-accessible teaching, although it does not list specific competencies. The South African disability framework also includes implementation and monitoring provisions. It indicates that:

[*C*]ommon indicators will be developed and agreed upon to measure and report on the progress in the implementation of the *Strategic Policy Framework on Disability*. Important is that this mechanism will also be used to make recommendations on how to improve the implementation of the inclusion of people with disabilities in PSET institutions. (South Africa. DHET, 2018, p. 73)

This offers a mechanism through which communication access indicators could be added.

In Malawi, the *National Education Policy* states that capacity development in higher education should be enhanced and includes strategies to ‘develop capacity of academic staff and directorate staff’ (Malawi. MoEST, 2016, p. 66). The *National Education Sector Investment Plan* similarly calls for capacity in ‘pedagogical skills for lecturers in Higher Education institutions’ (Malawi. MoE, 2020, p. 46). The Malawi *National Education Sector Investment Plan* also contains a stronger communication-related statement in the inclusive education section, calling for promotion of ‘sign language, braille and communication skills of teachers’ and improved teacher skills in maintaining assistive devices (Malawi. MoE, 2020, p. 50). This is relevant to students with communication disorders, although the provision is not framed specifically for higher education lecturers.

Overall, implementation mechanisms exist across policies, but the focus on communication-disorders remains limited. Policies call for staff development, accessible environments, ICT access and inclusivity, but do not define lecturer competencies in inclusive instructional communication. Such competencies would include clear speech, use of visual supports, accessible feedback, captioned content, communication-flexible assessment, sign language coordination, AAC awareness and referral pathways to communication support services.

### Comparative synthesis

The analysis of policy documents shows that South Africa has a more advanced policy architecture for disability inclusion, post-school education transformation and higher education language planning. Malawi has broad education and ICT policy commitments, a recent disability rights statute with strong communication definitions, and higher education standards that address physical access and learning resources. Across both countries, policies provide useful foundations for inclusive instructional communication, but they do not fully operationalise it (at policy level) for students with communication disorders. South Africa offers stronger policy mechanisms through its post-school disability framework, while Malawi’s *Persons with Disabilities Act, 2024* provides a strong legal definition of accessible communication, including AAC and sign language. The main shared gap is that communication access is not translated into detailed higher education instructional requirements. The findings therefore provide indicative policy insights. They show that national policy and legal frameworks support inclusivity in principle, but require more specific guidance on communicative access in teaching, learning, assessment and digital education for students with communication disorders.

## Discussion

The findings of this study show that inclusive instructional communication is present in South African and Malawian policy and legal frameworks, but mostly as an implied policy concern. It appears through related concepts such as inclusivity, accessibility, multilingualism, ICT access, disability rights, reasonable accommodation and staff development. However, it is rarely named, defined or operationalised as a higher education requirement for students with communication disorders. This is the central argument emerging from the analysis: policy and legal frameworks support inclusion as a broad commitment, but they do not sufficiently specify the communicative conditions through which students with hearing, speech, language and cognitive-communication disorders can participate fully in teaching, learning, assessment and academic life. Inclusion in higher education is more than admission, physical access or general disability support (Zhou, 2023). From an instructional communication perspective, participation depends on how knowledge is communicated, mediated, interpreted and assessed. Instructional communication research has shown that teaching and learning are shaped through clarity, feedback, interaction, relational engagement and meaning-making (Goodboy, 2018; Houser, 2019; Sellnow et al., 2015). But, when viewed from the position of students with communication disorders, these communicative processes are not merely pedagogical techniques; they are conditions of epistemic access. A student who cannot hear rapid spoken instruction, follow an uncaptioned lecture, use AAC during a seminar, access sign language interpretation or respond through a flexible assessment mode is not simply experiencing an individual difficulty. Such a student is encountering a policy and institutional environment in which communication has not been adequately designed as a condition of inclusivity.

The findings therefore show that the removal of barriers should include communicative barriers. Inclusive education, as Liasidou (2012) argued, requires educational systems to adapt to learner diversity instead of requiring learners to fit fixed institutional arrangements. In this study, such adaptation means that higher education policy should not only affirm disability inclusion but also identify the communication practices necessary for inclusion to become real in classrooms, lecture halls, laboratories, tutorials, online platforms and assessment spaces. For students with communication disorders, this includes captioning, transcription, assistive listening systems, accessible multimedia, multimodal teaching materials, sign language interpreting, AAC-compatible participation, flexible oral assessment alternatives and lecturer training in communication-accessible pedagogy. Without these mechanisms, inclusion remains ethically attractive but practically incomplete.

A critical realist immanent critique (Bhaskar, 2016) helps to make sense of this incompleteness. At the empirical level, which corresponds with Objective 1, the policy texts make visible commitments to inclusivity, equity, accessibility, multilingualism and disability support. These are not insignificant. South Africa’s policy environment gives relatively stronger attention to language, post-school disability inclusion and institutional transformation, while Malawi’s framework reflects broader education sector inclusion and a strengthened rights-based disability orientation. The empirical policy surface therefore suggests that both countries recognise inclusivity as a public good and as a policy obligation. However, critical realism cautions against taking visible policy statements as equivalent to social transformation. What is stated in policy is only one layer of reality (Stavrianos, 2025). At the real level, which corresponds with Objective 2, the analysis asks whether the policies identify generative mechanisms capable of producing inclusive instructional communication for students with communication disorders. This is where the main weakness appears. Some mechanisms are present, including provisions on reasonable accommodation, assistive devices, staff development, ICT access, disability rights, special needs education and language support. However, these mechanisms are too general to guide communication-accessible teaching and learning. They do not consistently explain how lecturers should adapt spoken instruction, how universities should support students who use AAC, how sign language should be integrated into higher education teaching, how digital platforms should be made accessible to students with communication difficulties or how assessments should accommodate speech, language, auditory and cognitive-communication barriers. In critical realist terms (Bhaskar, 2008), the policies contain inclusion-oriented intentions, but they do not sufficiently articulate the mechanisms that could transform those intentions into communicative participation. At the actual level, which corresponds with Objective 3, the comparison between South Africa and Malawi shows that both countries produce similar policy effects despite different policy trajectories. South Africa has more developed higher education language and disability structures, while Malawi has a wider education-sector approach and a strong legal recognition of communication within disability rights.

Nevertheless, in both cases, the actual policy problem is the same: inclusive instructional communication is not fully operationalised for students with communication disorders. The comparison therefore does not simply show that one country is stronger than the other. Instead, it shows that both contexts reveal a deeper Southern African policy challenge: inclusion is mostly expressed as access to the institution, but not sufficiently as access to the communicative processes through which knowledge is produced, shared and evaluated. This point is also decolonial. Calls to decolonise higher education in Africa (Mampane et al., 2018; Ndlovu-Gatsheni, 2018) require attention not only to curriculum content but also to the languages, modes and communicative practices through which knowledge is actualised (Mawonga & De Souza, 2026). Heleta (2016) argued that dominant colonial pedagogical models advance particular forms of language, interaction and knowledge production, thereby marginalising alternative communicative practices and epistemologies. In multilingual and postcolonial societies such as South Africa and Malawi, communication is never apolitical. It is shaped by histories of colonial language hierarchy, institutional authority and unequal access to academic voice. Nkomo (2021) therefore rightly cautioned that:

[*T*]here are no quick fixes to this question [of language and the role that higher education plays]; otherwise, oversimplifying it renders most projects and activities geared towards redressing the sociolinguistic legacy of colonialism and apartheid superficial, and indeed a mockery to the most democratic language policy and the struggles that made it possible. This position is inspired by the experiences of the transformation of European vernaculars into not only powerful but also imperial languages such as English and French, as well as the revolutionary growth of Afrikaans. This, however, should not be interpreted as an endorsement of the imperialistic and racially discriminatory history associated with those languages (p. 543).

Nkomo’s argument is crucial for interpreting the findings. If language transformation has no quick fixes, then inclusive instructional communication also cannot be solved through simple policy declarations. It requires sustained attention to the sociolinguistic, pedagogical, technological and disability-related conditions under which communication occurs. For students with communication disorders, the dominance of spoken, rapid, text-heavy and monolingual academic communication can reproduce exclusion even where institutions formally affirm inclusion. Decolonisation therefore demands more than replacing one language with another or adding multilingual aspirations to policy (Heugh, 2013, 2015; Steyn & Reygan, 2017). It requires rethinking the modes of expression, comprehension, silence, gesture, signing, assisted communication and embodied participation and how they are recognised in higher education.

The findings also show why disability inclusion and multilingualism should not be taken as separate policy concerns. In practice, students with communication disorders may experience layered barriers: auditory barriers, speech perception challenges, language processing difficulties, communication fatigue, limited access to spoken information, inaccessible digital content and exclusion from oral participation. These barriers may become more complex in multilingual classrooms where English or another dominant language functions as the medium of instruction. Thus, a student may not only face learning difficulties by inaccessible communication systems but also marginalised by language hierarchies that prefer particular accents, speeds, registers and forms of academic expression. This supports the argument by Maguvhe and Masuku (2023) that African inclusive education challenges require African epistemological responses. Such responses should take seriously the intersection of disability, multilingualism, technology and colonial histories of communication. The study therefore pushes the limits of instructional communication theory beyond general concerns with effective teaching. In the contexts examined, instructional communication should be understood as a justice-oriented practice. Clarity, feedback, immediacy and interaction are important, but they become insufficient when not connected to communicative accessibility. A lecture may be clear to hearing students and inaccessible to deaf students. A discussion may be engaging for fluent speakers and exclusionary for students with speech or language disorders. Digital or ICT-mediated learning may expand access for some students and exclude others when videos are uncaptioned, platforms are incompatible with assistive technologies or materials are not provided in accessible formats (De Souza & Kathumba, 2026). Inclusive instructional communication therefore requires a shift from communication as pedagogical efficiency to communication as an ethical and epistemic condition of participation.

The findings also have implications for communication disorders as a field. Communication disorders should not be understood only in clinical or individual terms. This study shows that communication disorders are also shaped by policy environments, institutional systems and pedagogical arrangements. Higher education policy can either enable or restrict communicative participation. Where policy does not specify captioning, sign language, AAC, accessible assessment and lecturer competence, it leaves students dependent on institutional discretion. In this sense, policy itself becomes part of the communication support system. It can either produce enabling conditions or reproduce silence around the needs of students with communication disorders. Practically, the findings suggest that higher education policy in both South Africa and Malawi should also include communication-specific inclusion. Policies should define inclusive instructional communication and require institutions to provide communication-focused lecturer development, accessible digital learning environments, captioning and transcription for live and recorded teaching, sign language interpreting where needed, AAC-compatible participation options, multimodal teaching resources, accessible assessment alternatives, classroom acoustic considerations and collaboration among disability units, lecturers, speech-language therapists, audiologists and assistive technology specialists. These are not additional benefits; they are mechanisms through which the right to participate in higher education becomes meaningful.

Overall, the study shows that inclusive instructional communication is a missing policy bridge between inclusive education, communication disorders and decolonial transformation. South Africa and Malawi both express strong commitments to equity and inclusivity, but these commitments remain underdeveloped where communication is concerned. Through a critical realist perspective (Bhaskar, 2008, 2016; Lawani, 2021), the empirical policy language is visible, the real mechanisms are partial, and the actual comparative pattern reveals a shared gap between inclusive aspiration and communicative participation. The philosophical implication is that inclusion is not achieved when students are merely present in higher education. Inclusion is achieved when students can access, interpret, question, contribute to and be recognised in the communicative life of the university.

## Conclusion

This study examined how inclusive instructional communication in higher education is conceptualised in policy and legal frameworks in South Africa and Malawi. The findings showed that both countries express commitments to equity, access, disability inclusion, multilingualism and ICT development. These commitments provide an important foundation for inclusive higher education. The analysis also showed that inclusive instructional communication for students with communication disorders remains insufficiently specified. Policies refer to disability inclusion, special needs access, reasonable accommodation, assistive technology, multilingualism and ICT access, but they do not provide guidance on how teaching and learning communication should be adapted for students with hearing, speech, language, auditory processing or cognitive-communication disabilities. These findings are indicative policy insights because the study analysed national policy documents and did not examine institutional policies, lecturer practices or student experiences. Future research should investigate how universities in both countries implement communication accessibility and how students with communication disorders experience lectures, tutorials, assessments, online learning and student support services. The study recommends that higher education policies in South Africa and Malawi should explicitly include inclusive instructional communication standards. These standards should address captioning, sign language support, AAC access, multimodal teaching, accessible digital learning, communication-accessible assessment and lecturer development in communication-disorders-informed pedagogy. This would strengthen the translation of inclusion commitments into teaching and learning practices that support communicative participation for students with communication disorders.
